# The Challenges of Using Oropharyngeal Samples To Measure Pneumococcal Carriage in Adults

**DOI:** 10.1128/mSphere.00478-20

**Published:** 2020-07-29

**Authors:** Laura K. Boelsen, Eileen M. Dunne, Katherine A. Gould, F. Tupou Ratu, Jorge E. Vidal, Fiona M. Russell, E. Kim Mulholland, Jason Hinds, Catherine Satzke

**Affiliations:** a Infection and Immunity, Murdoch Children’s Research Institute, Royal Children’s Hospital, Parkville, Victoria, Australia; b Department of Paediatrics, The University of Melbourne, Parkville, Victoria, Australia; c Institute for Infection and Immunity, St. George’s University of London, London, United Kingdom; d BUGS Bioscience, London Bioscience Innovation Centre, London, United Kingdom; e Ministry of Health and Medical Services, Suva, Fiji; f Department of Microbiology and Immunology, University of Mississippi Medical Center, Jackson, Mississippi, USA; g London School of Hygiene & Tropical Medicine, London, United Kingdom; h Department of Microbiology and Immunology, The University of Melbourne at the Peter Doherty Institute for Infection and Immunity, Parkville, Victoria, Australia; Escola Paulista de Medicina/Universidade Federal de São Paulo

**Keywords:** PCR, *Streptococcus pneumoniae*, carriage, genotypic, identification, nasopharyngeal, oropharyngeal, serotyping

## Abstract

Streptococcus pneumoniae (the pneumococcus) carriage is commonly used to measure effects of pneumococcal vaccines. Based on findings from culture-based studies, the World Health Organization recommends both nasopharyngeal (NP) and oropharyngeal (OP) sampling for detecting adult carriage. Given evidence of potential confounding by other streptococci, we evaluated molecular methods for pneumococcal identification and serotyping from 250 OP samples collected from adults in Fiji, using paired NP samples for comparison. Samples were screened using *lytA* quantitative PCR (qPCR), as well as pneumococcal identification and serotyping conducted by DNA microarray. A subset of OP samples were characterized by latex sweep agglutination and multiplex PCR. Alternate qPCR assays (*piaB* and *bguR*) for pneumococcal identification were evaluated. The *lytA* qPCR was less specific and had poor positive predictive value (PPV) in OP samples (88% and 26%, respectively) compared with NP samples (95% and 64%, respectively). Using additional targets *piaB* and/or *bguR* improved qPCR specificity in OP, although the PPV (42 to 53%) was still poor. Using microarray, we found that 102/107 (95%) of OP samples contained nonpneumococcal streptococci with partial or divergent complements of pneumococcal capsule genes. We explored 91 colonies isolated from 11 OP samples using various techniques, including multiplex PCR, latex agglutination, and microarray. We found that nonpneumococcal streptococci contribute to false positives in pneumococcal serotyping and may also contribute to spurious identification by qPCR. Our results highlight that molecular approaches should include multiple loci to minimize false-positive results when testing OP samples. Regardless of method, pneumococcal identification and serotyping results from OP samples should be interpreted with caution.

**IMPORTANCE** Streptococcus pneumoniae (the pneumococcus) is a significant global pathogen. Accurate identification and serotyping are vital. In contrast with World Health Organization recommendations based on culture methods, we demonstrate that pneumococcal identification and serotyping with molecular methods are affected by sample type. Results from oropharyngeal samples from adults were often inaccurate. This is particularly important for assessment of vaccine impact using carriage studies, particularly in low- and middle-income countries where there are significant barriers for disease surveillance.

## Abstract (precis)

Streptococcus pneumoniae (the pneumococcus) is a significant global pathogen. Accurate identification and serotyping are vital. In contrast with World Health Organization recommendations based on culture methods, we demonstrate that pneumococcal identification and serotyping with molecular methods are affected by sample type. Results from oropharyngeal samples from adults were often inaccurate. This is particularly important for assessment of vaccine impact using carriage studies, particularly in low- and middle-income countries where there are significant barriers for disease surveillance.

## INTRODUCTION

The Gram-positive bacterium Streptococcus pneumoniae (the pneumococcus) causes a range of diseases, including otitis media, pneumonia, and meningitis. Pneumococci are a significant cause of morbidity and mortality worldwide, particularly in young children and older adults (1, 2). Colonization, and subsequent carriage, of pneumococci in the upper airways (particularly the nasopharynx and oropharynx) is considered a prerequisite for pneumococcal disease and transmission (3). Carriage of pneumococci in healthy individuals is generally asymptomatic (3, 4). Pneumococcal conjugate vaccines (PCVs) targeting the pneumococcal polysaccharide capsule (current pediatric formulations contain 10 and 13 serotypes) have substantially reduced pneumococcal disease caused by vaccine-type pneumococci (5). In some settings, a subsequent increase in disease caused by nonvaccine serotypes has partially offset the benefits of PCVs (6, 7). PCVs also reduce carriage of vaccine-type pneumococci (8, 9). This, in turn, reduces transmission of pneumococci to unvaccinated individuals and therefore protects unvaccinated age groups within a population through indirect (or herd) effects.

Carriage studies are used to measure direct and indirect effects of pneumococcal vaccination in a population, particularly in low- or middle-income settings that lack robust disease surveillance systems (10). In children, pneumococci are primarily found in the nasopharynx, and so carriage is normally determined by testing nasopharyngeal (NP) swab specimens (11, 12). In contrast, pneumococcal carriage in adults is more evenly distributed between the nasopharynx and the oropharynx (13–16). Therefore, the World Health Organization (WHO) recommends collecting both oropharyngeal (OP) and NP samples for the detection of pneumococcal carriage in adults (11, 12). However, the 2013 recommendations were based on carriage studies using culture-based methodology, where the sensitivity of sampling the nasopharynx alone ranged between 58% and 81% compared with sampling both sites. The WHO highlighted that further research was needed to ascertain whether this recommendation is appropriate when using molecular techniques, which are becoming more common globally (11). More recently, two studies have compared NP and OP sampling in adults and adolescents for the detection of pneumococci using molecular methods (17, 18). Both studies concluded that sampling the oropharynx was superior to sampling the nasopharynx. A recent review examining studies of upper respiratory tract carriage of S. pneumoniae in adults, including those that utilized molecular methods, also encourages the sampling of other respiratory sites, such as OP and/or saliva, in addition to NP sampling (19).

However, there is some evidence that the use of molecular methods, particularly for serotyping of S. pneumoniae in OP samples, may yield false-positive results (20, 21). This may be particularly problematic in serotyping of S. pneumoniae in OP samples due to the presence of capsule biosynthesis genes in nonpneumococcal species similar to pneumococcal capsule loci. In this study, we examined whether molecular methods can be used to accurately identify and serotype pneumococci in adult OP samples. To do this, we used molecular methods for identification and serotyping on 250 paired adult NP and OP swabs collected as part of a vaccine evaluation study in Fiji (22). We assessed whether the use of multiple quantitative PCR (qPCR) targets could improve molecular detection of pneumococci from OP samples, and investigated how the presence of nonpneumococcal streptococci may confound molecular detection and serotyping of pneumococci.

## RESULTS AND DISCUSSION

The aim of our study was to examine the performance of molecular methods for identification and serotyping of pneumococci in adult OP samples. To do this, we evaluated the detection of pneumococci applying molecular methods to 250 paired NP and OP samples, collected from the same participant but stored and processed individually. A subset of OP samples and isolates was further explored to investigate potential false-positive results caused by nonpneumococcal streptococci.

### S. pneumoniae identification in NP and OP samples.

Samples were initially screened for pneumococci using DNA extracted directly from skim milk-tryptone-glucose-glycerol (STGG) medium using *lytA* qPCR, with samples classified as positive (cycle threshold value [*C_T_*], <35), equivocal (*C_T_*, 35 to 40), or negative (no *C_T_*) (Table 1). Forty-three (17%) of the 250 OP samples screened were either *lytA* positive (9/250 [4%]) or equivocal (34/250 [14%]), while 33 (13%) of the 250 NP samples tested were either *lytA* positive (14/250 [6%]) or equivocal (19/250 [8%]). All *lytA*-positive and equivocal samples were tested for the presence of pneumococci using DNA microarray following culture enrichment on selective agar (23).

**TABLE 1:** Pneumococcal carriage results obtained from paired nasopharyngeal and oropharyngeal swabs^*a*^

*lytA* qPCR result	Microarray result	NP^*b*^ samples (*n* = 250)	OP^*c*^ samples (*n* = 250)
Positive (*C_T_*, <35)	Positive	13	7
	Negative	0	2
	No growth^*d*^	1	0
Equivocal (*C_T_*, 35–40)	Positive	7	3
	Negative	0	30
	No growth^*e*^	12	1
Negative (no *C_T_*)		217	207

a Samples were screened by *lytA* quantitative real-time PCR, and positive and equivocal samples were further tested by culture on selective agar and DNA microarray.

b NP, nasopharyngeal.

c OP, oropharyngeal.

d One NP sample was nonculturable and therefore not tested by microarray.

e Equivocal samples with no growth were considered negative for pneumococci (54).

For NP samples, microarray detected pneumococci in 13/14 (93%) *lytA*-positive samples and 7/19 (37%) *lytA*-equivocal samples. In contrast, microarray detected pneumococci in 7/9 (78%) *lytA*-positive OP samples, compared with only 3/34 (9%) *lytA*-equivocal OP samples. The proportion of *lytA*-equivocal samples that were found to be true positives for pneumococci was higher for NP samples than for OP samples (*P* = 0.025), suggesting that the *lytA* gene is a poor target for the detection of pneumococci in adult OP samples. Our findings are consistent with a study examining carriage in Kenyan adults using 40 combined NP/OP samples, in which the real-time *lytA* PCR positivity rate (60%) was much higher than what could be confirmed using culture (12.5%) (20).

When cultured on selective media, OP samples typically produced a greater diversity of colony morphologies (and therefore may be more likely to contain a number of different gentamicin-resistant species [see Fig. S1 in the supplemental material]) than NP samples. Consistent with this, pneumococci were only detected by microarray in 10/42 (24%) of OP samples that had bacterial growth on gHBA (horse blood agar plates containing 5 μg/ml of gentamicin) plates, compared with 20/20 (100%) NP samples (two additional NP samples were not tested by microarray, as they lacked α-hemolytic growth). OP samples also had a greater density of bacterial growth on gHBA plates than NP samples: 41/42 (98%) of OP samples had growth on a 1:100 dilution plate, compared with 13/22 (59%) of NP samples (*P* < 0.001), consistent with a study with Dutch adults (17).

10.1128/mSphere.00478-20.1 FIG S1 Example of bacterial growth on a gHBA plate for a nasopharyngeal sample (a) compared with an oropharyngeal sample (b). Download FIG S1, DOCX file, 0.7 MB. Copyright © 2020 Boelsen et al. 2020 Boelsen et al. This content is distributed under the terms of the Creative Commons Attribution 4.0 International license.

### OP sample characterization.

We hypothesized that the presence of nonpneumococcal streptococci in the oropharynx may contribute to the *lytA* signal and may lead to false identification of pneumococci. We therefore sought to confirm the presence of nonpneumococcal streptococci using microarray which, though not designed specifically for identification of individual streptococci, can confirm the presence of streptococcal species. We tested 30 OP samples using microarray (5 of which were *lytA* positive and 25 *lytA* equivocal) and found that while only 5 (17%) contained pneumococci, all contained complex mixtures of nonpneumococcal species, including Streptococcus salivarius, Streptococcus mitis, Streptococcus oralis, Streptococcus infantis, and, less commonly, Streptococcus parasanguinis, Streptococcus anginosus, and Streptococcus sanguinis. The detection of streptococci by microarray also suggests that the reasons for discrepancies between *lytA* qPCR and microarray in pneumococcal detection are not due to poor sensitivity of microarray. While S. pneumoniae has distinct *lytA* alleles compared with the mitis group, there is evidence that *lytA* qPCR can both misidentify commensal streptococci as pneumococci and fail to identify pneumococci (24, 25). Genetic exchange with streptococci has played a major role in evolution of S. pneumoniae, and many S. pneumoniae virulence genes are also present in S. mitis, S. oralis, and *S. infantis* (26). As bacterial diversity in the oropharynx is higher than in the nasopharynx (27), there is greater opportunity for genetic exchange between streptococci. This genetic exchange, particularly within members of the mitis group streptococci, means that the reliance on one single loci for identification may result in erroneous species identification (28).

### Alternative qPCR-based S. pneumoniae identification in OP samples.

Next, we aimed to improve qPCR-based identification of pneumococci in OP samples by evaluating alternative target genes, which could be potentially used alone or in combination with *lytA*. We assessed the seven potential targets (Tables S2 and S3) by conventional and real-time PCR against a set of 30 reference isolates (6 S. pneumoniae and 24 nonpneumococcal streptococcal isolates) (Tables S4 and S5). Based upon the results, the genes *bguR* and *piaB* were further tested.

The *bguR* and *piaB* qPCR assays were applied to DNA extracted from 250 OP samples, which had been previously tested with *lytA* qPCR. One hundred forty-two samples were negative for all three targets. One hundred eight samples were positive or equivocal for either *bguR*, *piaB*, and/or *lytA* and were subsequently cultured on gHBA plates and analyzed by microarray. Based on microarray results, the gold standard for pneumococcal identification in our study by detection of multiple species-specific genes, 11 (10%) out of 108 samples contained S. pneumoniae. Note that the matched NP sample was also positive for pneumococci for two of these 11 OP samples; therefore, by using OP samples, nine additional pneumococcal carriers were detected.

The three qPCR targets were evaluated for sensitivity, positive predictive value (PPV), and specificity individually and in combination using a *C_T_* of <40 as a cutoff for positivity (Table 2).

**TABLE 2:** Evaluation of *lytA*, *bguR*, and *piaB* qPCR (alone and in combination) for detection of pneumococci in 250 OP samples^*a*^

Target gene(s)	No. of:						
	True positives	False positives	False negatives	True negatives	PPV (%)^*b*^	Specificity (%)	Sensitivity (%)
*lytA*	10	29	1	210	26	88	91
*bguR*	10	68	1	171	13	72	91
*piaB*	10	20	1	219	33	92	91
*lytA* + *bguR*	10	14	1	225	42	94	91
*lytA* + *piaB*	9	11	2	228	45	95	82
*bguR* + *piaB*	9	10	2	229	47	96	82
*lytA* + *bguR* + *piaB*	9	8	2	231	53	97	82

a A positive is defined as any *C_T_* of <40.

b Positive predictive value (PPV), specificity, and sensitivity were compared to DNA microarray, the study gold standard for pneumococcus-positive samples.

Individually, none of the targets performed well: *piaB* had 33% PPV and 92% specificity, *lytA* had 26% PPV and 88% specificity, and *bguR* had 13% PPV and 72% specificity. All three targets had one false negative (to give a sensitivity of 91%). When two targets were combined, the PPV and specificity improved (ranging from 42 to 47% and 94 to 96%, respectively), although when *piaB* was used with *lytA* or *bguR* there were two false negatives, reducing the sensitivity of these combinations to 82%. Combining all three targets further improved both PPV (53%) and specificity (97%) but yielded lower sensitivity (82%) than for single targets. It should be noted that the PPV of all approaches and the specificity of most approaches investigated were inferior to the use of *lytA* in NP samples (64% PPV and 95% specificity). Our findings were in contrast to those of a recent study exploring the use of *lytA*, *piaB*, and SP2020 (*bguR*) assays for the detection of pneumococci (29). When tested on 80 OP samples, there were few inconsistencies between assays and no apparent issues with the *lytA* assay, and combining assays eliminated false positives. The reasons for the discrepancies between studies is unclear.

The primary aim of this study was to evaluate targets for use in a screening assay, and therefore, we focused primarily on specificity and PPV. Any sample that was negative by all three targets was deemed a true negative. For each individual assay, there was a subset of samples that were negative for that assay and were tested further (69, 30, and 78 for *lytA*, *bguR*, and *piaB*, respectively). However, one limitation of our study is that we lacked a complete measure of false negatives and therefore did not fully evaluate sensitivity and negative predictive value.

If these assays were used for identification rather than detection of pneumococcus-positive adult OP samples, a more stringent cutoff would be more appropriate. When we applied a cutoff *C_T_* of <35 to our OP samples, *lytA* was the best target (99% specificity, 78% PPV, and 64% sensitivity) and the use of multiple targets was not appropriate (all other target/s had ≤36% sensitivity) (Table S6). We also considered a sequential screening approach whereby only those samples with a *C_T_* of <40 for one target would be further tested by other targets, which gave 37 to 41% PPV, 93 to 95% specificity, and 82 to 91% sensitivity (Table S7). We also explored the possibility that false-positive samples could be identified through differences in signal intensity between two targets; however, no clear differences were observed in positive samples versus negative samples for any target combination. Although we had only a small number of pneumococcus-positive OP samples, our results indicate that qPCR may be unreliable for accurate detection of pneumococci in OP samples even when multiple targets are used.

### S. pneumoniae serotyping in NP and OP samples.

**FIG 1:**
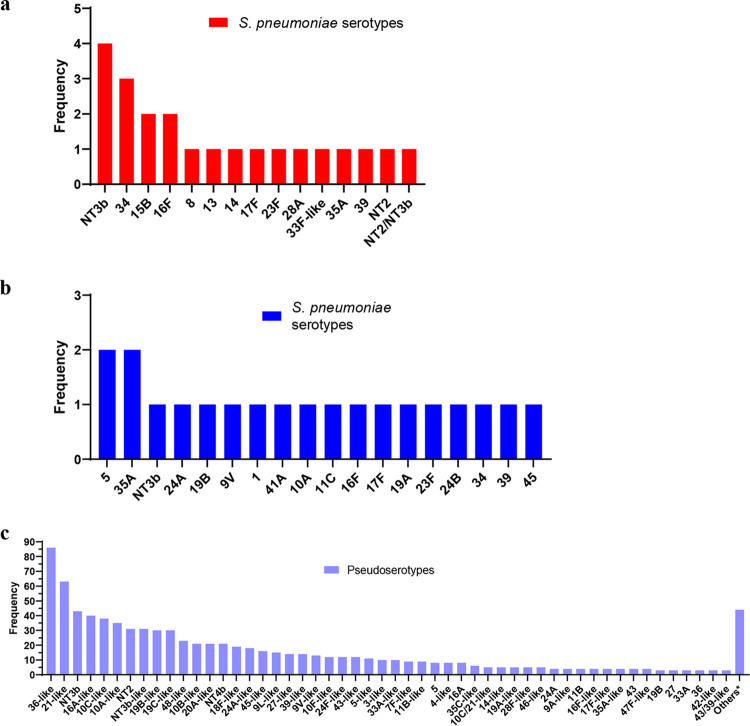
(a) Frequency of pneumococcal serotypes detected in NP samples. (b and c) Pneumococcal capsular genes detected in OP samples from S. pneumoniae (serotypes) (b) or other species (pseudoserotypes) (c). *, others include 9V, 9L, 18A-like, 18C-like, 20-like, 33F-like, 37, 41F-like, 47A, NT3a, and NT4a (all *n* = 2) and 1, 41A, 7A-like, 7F, 9A, 9N-like, 10F, 13-like, 17A-like, 17-like, 20A, 22A, 24B-like, 28A-like, 32F-like, 33B-like, 35B, 35F-like, 40, 41A-like, 46, and 48 (all *n* = 1).

Next, we conducted molecular serotyping of pneumococci from NP and OP samples using DNA microarray. The microarray detects multiple targets for each of the pneumococcal capsule genes in order to assign a serotype, and it is the best method for detecting multiple serotypes in NP samples from children owing to its high sensitivity and PPV (95.8% and 93.9%, respectively) (23). Serotyping of NP samples from adults using microarray was relatively straightforward (Fig. 1a), with only one (18/20 [90%]) or two (2/20 [10%]) serotypes detected in each sample. Additionally, all samples were clearly positive for pneumococci and no other streptococci were detected.

In contrast, OP samples were highly complex and challenging to serotype, with the presence of partial or divergent sets of capsule genes that were likely to be present in nonpneumococcal species, here referred to as pseudoserotypes. Serotyping was attempted on 108 OP samples, with a total of 876 serotypes (*n* = 20) and pseudoserotypes (*n* = 856) detected (Fig. 1b and c). Only one OP sample had no growth on blood agar plates and was therefore unable to be further tested. Of the 107 OP samples, 55 (51%) had 10 serotypes and/or pseudoserotypes detected, only 5 samples had no serotypes or pseudoserotypes (streptococci were detected in all 5, so this was not due to an error in the microarray) and no OP samples had only pneumococci and pneumococcal serotypes. The most common pseudoserotype was similar to serotype 36 (“36-like,” indicating that a partial or divergent set of serotype 36 pneumococcal capsule genes was detected) and was present in 83% (89/107) of OP samples.

### Additional characterization of OP samples.

We next investigated whether the presence of pseudoserotypes in nonpneumococcal streptococci in the OP samples could lead to false-positive serotyping results using other methods, and/or potentially produce capsule similar to true pneumococci. We selected 11 OP samples for this detailed analysis, representing a variety of their prior *lytA* qPCR and microarray serotyping results. For example, two samples were *lytA* positive but had no pneumococci detected by microarray, while another had a complete (or near complete) set of capsule genes for serotype 9V but had no pneumococci detected. Only 2 of the 11 samples were found to contain pneumococci using microarray. We tested the 11 OP samples with antibody-based and molecular-based serotyping methods (latex sweep agglutination and multiplex PCR [mPCR], respectively) (Table 3). When latex sweep agglutination was used, 6 of 11 samples had a serotype detected. For mPCR, all 11 samples had a serotype detected; however, for 2 samples, the *cpsA* pneumococcal control gene was absent. While all three methods (microarray, latex sweep agglutination and mPCR) detected pseudoserotypes, only microarray designated these as coming from nonpneumococcal species.

**TABLE 3:** Detailed microbiological analysis of 11 OP samples using *lytA* qPCR, microarray, latex sweep agglutination, and multiplex PCR

Sample ID	*lytA* qPCR result (*C_T_*)^*a*^	Microarray PathID^*b*^	Microarray serotyping^*c*^	Latex sweep agglutination	mPCR *cpsA* result^*d*^	mPCR serotyping^*e*^
FVEP-002-004	Equivocal (39.52)	SP-3/5	9A-like* (34%) + 7F-like* (26%) + 36-like* (17%) + NT4b* (17%) + 27-like* (6%)	4 + 9L + 36 + 15	Positive	7F/7A + 24F/24A/24B + 10F/10C/33C + 9N/9L
FVEP-002-418	Equivocal (38.48)	SP-3/5	NT4a* (53%) + 24A-like* (21%) + NT4b* (11%) + 43/39-like* (9%) + 16A-like* (3%) + 28A-like* (2%) + 4-like* (1%)	24A	Negative	33F/33A/37 + 35B + 24F/24A/24B + 4 + 10F/10C/33C + 35A/35C/42 + 9N/9L
FVEP-002-496	Equivocal (37.93)	SP-3/5	10C/21-like* (41%) + NT4a* (20%) + 36-like* (12%) + 39-like* (8%) + 16A-like* (8%) + 48-like* (4%) + 24A-like* (4%) + 45-like* (2%) + 19B-like* (1%)	NSD^*f*^	Positive	22F/22A + 24F/24A/24B + 10A + 10F/10C/33C + 20 + 13
FVEP-002-460	Equivocal (35.82)	SP-5/5	NT4b* (35%) + 10C/21-like* (29%) + 48-like* (11%) + 41A (7%) + 20-like* (5%) + 23F (4%) + 35A-like* (4%) + 5-like* (3%) + 16A-like* (2%)	NSD	Positive	6A/6B/6C/6D + 6C/6D + 22F/22A + 33F/33A/37 + 35B + 4 + 23F + 10A + 10F/10C/33C + 5 + 35A/35C/42 + 34 + 21 + 20
FVEP-002-002	Equivocal (37.79)	SP-3/5	NT4b* (46%) + 33A-like* (40%) + 9V (5%) + 18F-like (4%) + 35A-like (4%) + 19B-like (1%)	12 + 33 + 35B	Positive	6A/6B/6C/6D + 19A + 33F/33A/37 + 38/25F/25A + 35B + 10A + 10F/10C/33C + 35A/35C/42
FVEP-002-084	Equivocal (37.57)	SP-3/5	NT4b* (62%) + 36-like* (25%) + 5-like* (7%) + 19B-like* (5%) + 9N-like* (1%)	19B	Positive	4 + 5 + 9N/9L
FVEP-002-488	Equivocal (39.82)	SP-3/5	NT4b* (45%) + 48-like* (36%) + 19B-like* (11%) + 43/39-like* (8%)	NSD	Negative	38/25F/25A + 4 + 23F + 39 + 10F/10C/33C + 34 + 21 + 20 + 6A/6B/6C/6D
FVEP-002-078	Positive (28.44)	SP-3/5	NT4b* (26%) + 10C/21-like* (26%) + 7F-like* (26%) + 16A-like* (11%) + 45-like* (6%) + 9L-like* (5%)	35B	Positive	15A/15F + 7F/7A + 24F/24A/24 + 4 + 10F/10C/33C + 9N/9L + 21 + 20
FVEP-002-080	Positive (34.41)	SP-5/5	NT4b* (32%) + 48-like* (27%) + 24B (20%) + 19B-like* (13%) + 1 (8%)	19B	Positive	24F/24A/24B + 1 + 9N/9L + 13
FVEP-002-422	Positive (34.70)	SP-3/5	NT4b* (42%) + 10C/21-like* (42%) + 19C-like* (12%) + 16A-like* (4%)	NSD	Positive	15B/15C + 10F/10C/33C + 21 + 17F
FVEP-002-486	Equivocal (35.29)	SP-3/5	NT4b* (71%) + 10C/21-like* (17%) + 11B-like* (5%) + 16A-like* (5%) + 19C-like* (2%)	NSD	Positive	15A/15F + 10F/10C/33C + 35A/35C/42 + 9N/9L + 31 + 20

a *C_T_*, cycle threshold value for *lytA* qPCR (*C_T_* of < 35, positive; *C_T_* of 35 to 40, equivocal; no *C_T_*, negative).

b SP-5/5 result for the PathID component on microarray indicates a sample positive for S. pneumoniae; SP-4/5 or less indicates that S. pneumoniae is not present.

c Pseudoserotypes are marked by “*” indicating that the result is likely from a nonpneumococcal bacterial species and “-like” indicating a partial or divergent set of capsule genes.

d *cpsA* in mPCR is used as a positive control for pneumococci.

e Each sample also had additional mPCR products that did not correlate with an expected mPCR serotype product size that are not listed here.

f NSD, no serotype detected.

### OP isolate characterization.

**FIG 2:**
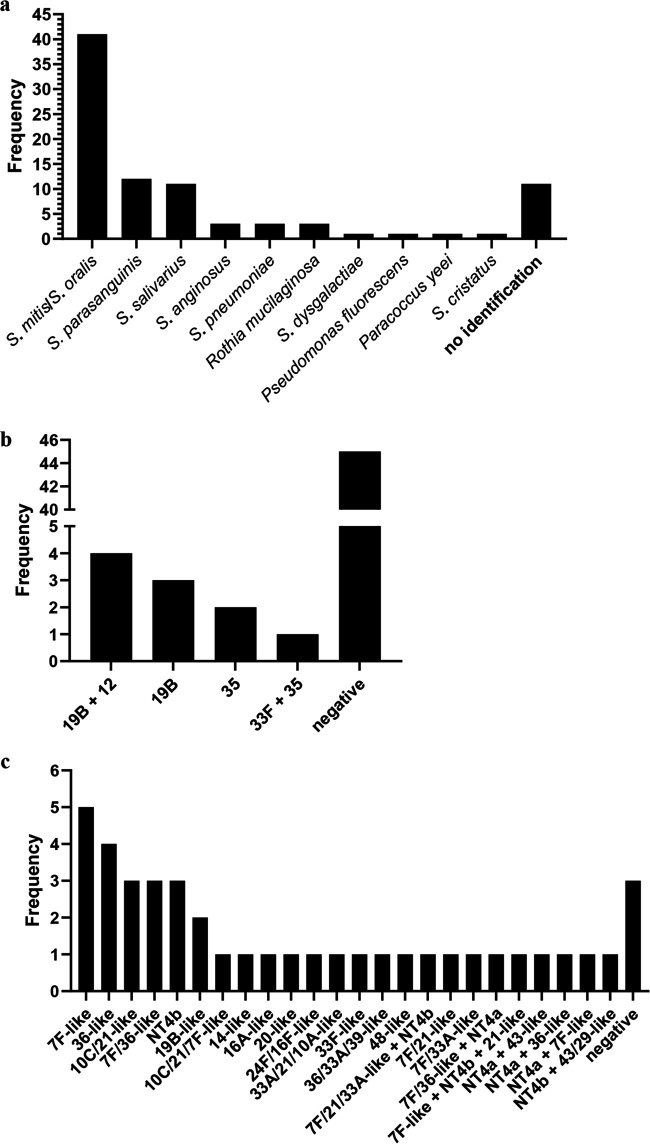
MALDI-TOF MS identification (a), latex agglutination (b), and microarray serotyping (c) results for 88 nonpneumococcal isolates subcultured from 11 OP samples. These data do not include results from 33 isolates that were not able to be tested by latex agglutination or from 47 isolates that were not tested by microarray for practical reasons.

We recultured these 11 OP samples on gHBA plates and isolated a representative of each colony morphology identified in the sample (*n* ≥ 5 for each sample). The resultant 91 isolates from these 11 OP samples were further characterized by the identification tests *lytA*, *bguR* and *piaB* real-time PCR, optochin susceptibility, bile solubility, and matrix-assisted laser desorption ionization–time of flight mass spectrometry (MALDI-TOF MS), as well as latex agglutination for serotyping (Table S8 and S9). An isolate was deemed S. pneumoniae if at least three out of four identification tests were indicative of S. pneumoniae*;* if only two tests were indicative of S. pneumoniae, DNA microarray was used to resolve identification. Only three of these isolates were identified as S. pneumoniae. Nearly half (41/88 [47%]) of the nonpneumococcal isolates were identified as S. mitis/S. oralis (not distinguished by MALDI-TOF MS), with *S. parasanguinis* (12/88 [14%]) and *S. salivarius* (11/88 [13%]) also common (Fig. 2a). For 11 of the 88 isolates, no identification was obtained by MALDI-TOF MS.

Serotyping by latex agglutination was attempted on all 91 isolates; however, 33 isolates did not emulsify in saline and so were not able to be tested. We did not identify a serotype for 45 isolates. A serotyping result was obtained for 10 of 55 nonpneumococcal isolates, with results shown in Fig. 2b. When the 10 isolates were serotyped by Quellung, none were positive, although some slight cell enlargement was observed for some isolates. The reasons for this were not explored, but it is plausible that the capsule polysaccharide produced may be thinner in these closely related species than pneumococcal capsule polysaccharide, or not covalently linked to the cell wall peptidoglycan, therefore making it difficult to observe the typical “swelling” in a Quellung reaction.

**FIG 3:**
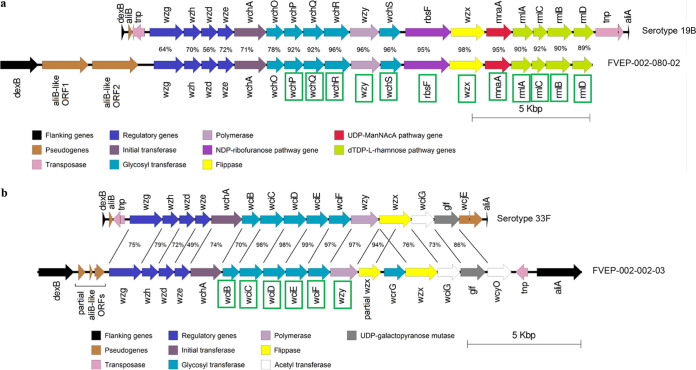
Comparison of the capsule gene locus for isolate FVEP-002-080-02 (a) with serotype 19B (GenBank accession no. CR931676.1) and the capsule gene locus for isolate FVEP-002-002-03 (b) with serotype 33F (GenBank accession no. AJ006986.1). Sequence identity (percent) between the two sets of genes is shown. Genes are categorized according to characteristics used by Bentley et al. (53). Green boxes around genes indicate that a gene was categorized as present by microarray serotyping.

To further elucidate the source of the spurious identification and serotyping results, a subset of isolates (*n* = 41) were serotyped by microarray. These included all isolates that were equivocal for *lytA* and positive for a serotype/serogroup in latex agglutination and at least one isolate for each unique MALDI-TOF MS result. Pseudoserotypes were detected for 38 of the 41 (93%) nonpneumococcal isolates tested, with results shown in Fig. 2c. We conducted whole-genome sequencing on two of the nonpneumococcal OP isolates (which had typed as 19B [*n* = 1] or 33F [*n* = 1] by latex agglutination) and compared the *cps* locus of these isolates with the *cps* locus of a corresponding true pneumococcal serotype. Isolate FVEP-002-080-02 (which serotyped as 19B by latex agglutination) was identified as *S. infantis* and had a full set of 19B capsule genes, although the first six genes shared <78% identity with the 19B reference isolate (Fig. 3a). Isolate FVEP-002-002-03, which serotyped as 33F using latex agglutination, was identified as Streptococcus oralis subsp. *tigurinus* (Fig. 3b). The *cps* region of this isolate was similar to that of 33F; however, two copies of *wzx* were present (one of which was truncated). A *wcrG* gene, which is normally found in serotype 10A, was also present. Excluding the truncated *wzx* gene and the *wcrG* gene, this *cps* locus was highly similar to the S. oralis subsp. *tigurinus* strain Az_3a *cps* locus (30), including the presence of the *wcyO* gene (which is most similar to that of serotype 21 and is not typically present in serotype 33F pneumococci).

### General discussion.

Our findings from testing the isolates confirm that the nonpneumococcal streptococci in OP samples can contribute to false positives for pneumococcal serotyping. Pseudoserotypes were found in all of the 38 nonpneumococcal streptococci isolates tested. Importantly, our results suggest that nonpneumococcal streptococci present in the oropharynx may be falsely identified as pneumococci and can also produce capsule similar to pneumococcal capsule. Consistent with this, several recent studies have reported closely related streptococci expressing capsular polysaccharide antigenically similar to pneumococcal capsule (30–32). Kilian et al. have suggested that the diversity of the *cps* locus emerged from unidirectional interspecies gene transfer from commensal streptococci to S. pneumoniae (33, 34). Recently, Streptococcus mitis strains that produce capsule immunologically consistent with pneumococcal serotype 1 have recently been reported (35), raising questions as to whether carriage of such strains would induce an immune response to pneumococci.

A key strength of our study was applying some of the best available methods for serotyping our OP samples. The use of multiple different methods also allowed us to demonstrate that specificity issues affect multiple types of serotyping methods and are not limited to one method alone. Samples were processed in batches that included both NP and OP samples and multiple extraction controls, thereby eliminating the possibility that underlying differences have emerged due to contamination or batch variability. A limitation of our study is that we used a culture enrichment step prior to pneumococcal identification using microarray, and it is possible that we may have missed some samples containing pneumococci. We chose microarray as a gold standard for pneumococcal identification in this study because it is not based on one single data point or gene sequence but includes targets over the entire genome, which provides greater confidence in results. It is plausible the presence of high-density nonpneumococcal species on culture plates for OP samples may mask the detection of pneumococci in downstream testing, or some pneumococci may be nonviable. However, Trzcinski et al. found that culture enrichment improved detection of pneumococci (17), and our overall findings are consistent with other studies which have shown the presence of pneumococcal gene homologs in other closely related streptococci (20, 21, 36). Despite our detailed investigation, we were also unable to definitively identify the source of spurious pneumococcal identification results. While we failed to find any nonpneumococcal isolate that was positive for the *lytA* gene, we did find 10 isolates which had some amplification. It is plausible that we may have missed those colonies which directly contributed to the *lytA* result. It is also possible that, cumulatively, the weak amplification in individual nonpneumococcal colonies contributes to equivocal results at a sample level.

We should also note that our adult participants were mostly female (93%) and under the age of 65 (98%) and therefore may differ from adult participants described in studies aimed at understanding disease in older adults.

Taken together, our results highlight the challenging nature of conducting a pneumococcal carriage study in adults using OP samples. The presence of other commensal streptococci in the oropharynx interferes with identification and serotyping of true pneumococci within an OP sample, regardless of whether culture-based or molecular serotyping methods are used. For adult pneumococcal carriage studies, approaches which utilize multiple targets or loci, or involve the isolation and careful identification of pneumococci, are necessary if OP samples are to be included, although the latter may be impractical for a large study.

Given the challenges of identifying and serotyping pneumococci using OP samples from adults, we caution against their routine use in carriage studies.

## MATERIALS AND METHODS

### Swab collection, transport, and storage.

**FIG 4:**
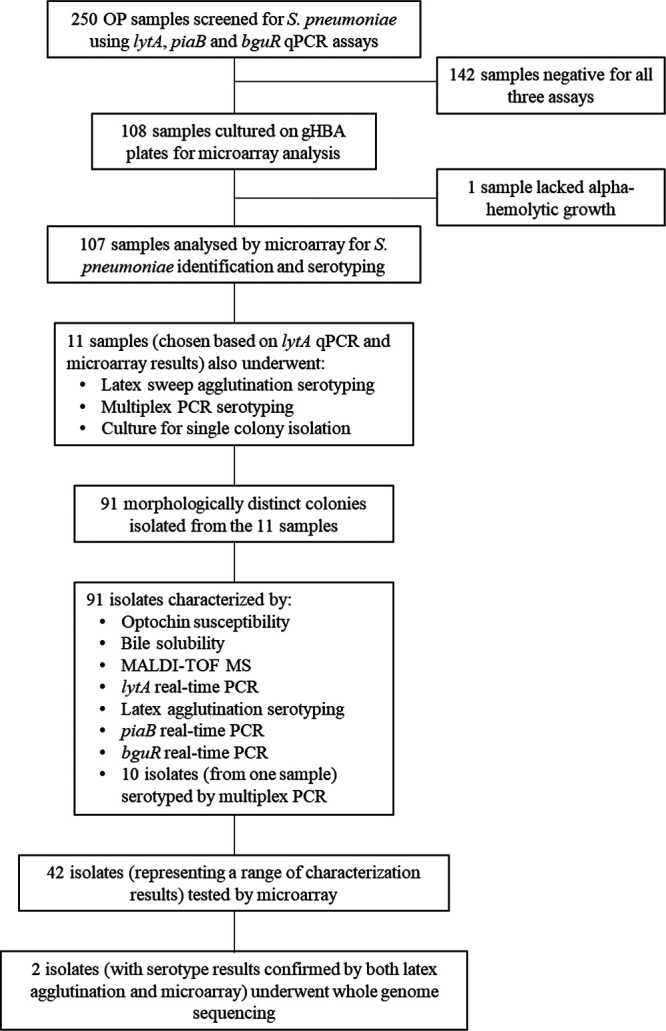
Experimental procedures for OP samples and isolates. gHBA, gentamicin horse blood agar.

An NP and OP swab were collected from 250 adult caregivers of children (adult caregivers) in 2012 as part of the Fiji New Vaccine Evaluation Project, described previously (22); see Table S1 for study participant characteristics. In brief, NP and OP specimens were collected concurrently by the same personnel using flocked nylon swabs (11, 12) and were otherwise handled in the same manner. Each swab was placed in 1 ml of skim milk-tryptone-glucose-glycerol (STGG) medium in an individual cryovial and transported in a cool box to the Fiji Centre for Communicable Disease Control in Suva, Fiji. On arrival at the laboratory, swabs were vortexed for 20 s prior to aliquoting and storage at −80°C within 8 h of collection, in line with WHO guidelines (11). Samples were shipped on dry ice to the Murdoch Children’s Research Institute in Melbourne, Australia, and stored at −80°C. For clarity, a flowchart showing the full set of experimental procedures conducted on OP samples and the derived isolates is presented in Fig. 4.

10.1128/mSphere.00478-20.2 TABLE S1 Characteristics of study participants. Download Table S1, DOCX file, 0.01 MB. Copyright © 2020 Boelsen et al. 2020 Boelsen et al. This content is distributed under the terms of the Creative Commons Attribution 4.0 International license.

### DNA extraction from swab samples.

DNA was extracted from 100 μl of STGG sample as previously described (22). Each extraction run contained approximately equal numbers of NP and OP samples and included STGG extraction controls that were checked for pneumococcal contamination using *lytA* qPCR.

### qPCR on swab samples.

The detection of S. pneumoniae by qPCR assays was performed on NP or OP swabs using DNA extracted as described above. Assays targeting the *lytA* and *piaB* genes were conducted as previously described (17, 37). An assay for the GntR-type transcriptional regulator gene *bguR* (38) (TIGR4 NCBI reference sequence, NC_003028; locus tag, SP_RS10220; old locus tag, SP_2020), also referred to as SP2020 (19, 29), used forward primer (5′-AGTTTGCCTGTAGTCGAATGA-3′), reverse primer (5′-TTTGAGCTGCCACGAGAG-3′) and the probe (5′-6-carboxyfluorescein [FAM]-AAACGTGGGCAGGGAACCTTTGTT-BHQ1-3′) concentrations of 300 nM, 100 nM, and 200 nM, respectively. No-template controls (blanks) were included in each qPCR run, and runs were balanced with approximately equal numbers of NP and OP samples. Samples were considered positive when the cycle threshold value (*C_T_*) was <35, equivocal when the *C_T_* was between 35 and 40, and negative when no *C_T_* value was obtained in 40 cycles.

### Pneumococcal identification and molecular serotyping by DNA microarray.

Samples that were either positive or equivocal for the *lytA*, *piaB*, or *bguR* gene were cultured for microarray analysis. Samples were analyzed by a pneumococcal molecular serotyping microarray (Senti-SP1.4/1.5; BUGS Bioscience) as previously described (23). Microarray analysis was performed following culture amplification on horse blood agar plates containing 5 μg/ml of gentamicin (gHBA; Oxoid brand, Thermo Fisher Scientific, Australia), and DNA extraction (for gHBA plates with α-hemolytic growth only) using the QIAamp 96 DNA QIAcube HT kit (Qiagen) or the QIAamp DNA minikit (Qiagen).

### Alternative S. pneumoniae target genes.

We considered alternative target genes to *lytA* for the detection of S. pneumoniae. Fifteen targets identified by a literature search and/or used in the S. pneumoniae StrepID component of the microarray were initially considered. We excluded 4 targets (*rpoA*, *sodA*, *tuf*, and *recA*) because they required sequencing of the PCR product and 4 targets (*ply*, *psaA*, spn9802, and 16S rRNA genes) because they have been reported to lack the specificity to distinguish between closely related species or the sensitivity to detect all pneumococci. Genes *piaA*, *piaB*, and *ulaA* were selected for further analysis as well as four additional genes used in the StrepID component of microarray (see Table S2 and S3). Targets were tested against a set of 30 reference isolates (6 S. pneumoniae and 24 nonpneumococcal streptococcal isolates) initially by PCR assays (see Table S4). Real-time qPCR assays for *lytA*, *piaB*, and *bguR* (described above) were also assessed against the reference isolates (see Table S5).

10.1128/mSphere.00478-20.3 TABLE S2 Potential S. pneumoniae-specific targets. Download Table S2, DOCX file, 0.02 MB. Copyright © 2020 Boelsen et al. 2020 Boelsen et al. This content is distributed under the terms of the Creative Commons Attribution 4.0 International license.

10.1128/mSphere.00478-20.4 TABLE S3 Primer sequences for *vanZ*, SP_0137, *bguR*, and *fucK*. Download Table S3, DOCX file, 0.01 MB. Copyright © 2020 Boelsen et al. 2020 Boelsen et al. This content is distributed under the terms of the Creative Commons Attribution 4.0 International license.

10.1128/mSphere.00478-20.5 TABLE S4 Results for potential pneumococcus-specific PCR assays tested against a panel of reference isolates. Download Table S4, DOCX file, 0.02 MB. Copyright © 2020 Boelsen et al. 2020 Boelsen et al. This content is distributed under the terms of the Creative Commons Attribution 4.0 International license.

10.1128/mSphere.00478-20.6 TABLE S5 Results for potential pneumococcus-specific real-time PCR assays tested against a panel of reference isolates. Download Table S5, DOCX file, 0.02 MB. Copyright © 2020 Boelsen et al. 2020 Boelsen et al. This content is distributed under the terms of the Creative Commons Attribution 4.0 International license.

### Isolation of bacteria from OP samples.

Samples were cultured directly on gHBA, and a representative of each colony morphology (including nonhemolytic and β-hemolytic colonies) for each sample was then subcultured onto HBA and incubated overnight (37°C, 5% CO_2_) with an optochin disc. All OP isolates (*n* = 91) were then tested for bile solubility (39). A laboratory strain of S. pneumoniae (PMP6, ATCC 6305) and S. mitis (PMP16) were used as controls for optochin sensitivity and bile solubility testing.

### DNA extraction on isolates.

A sweep of pure culture was inoculated into 100 μl of phosphate-buffered saline using a 10-μl sterile loop. Bacterial suspensions were pelleted at 7,380 × *g* for 10 min. Initially, DNA was extracted from all OP isolates using the QIAcube HT instrument (Qiagen). Prior to loading the instrument, pellets were resuspended in lysis buffer (20 mM Tris-HCl, 2 mM Na-EDTA, 1% [vol/vol] Triton X-100, 2 mg/ml of RNase A, and 20 mg/ml lysozyme) and incubated at 37°C for 60 min. Twenty microliters of 20-mg/ml proteinase K (Qiagen) and 200 μl of buffer AL (Qiagen) were added before incubation at 56°C for a further 30 min.

However, for many of the isolates (*n* = 52) there were issues during the extraction process resulting in poor-quality DNA. These isolates were extracted using the QIAamp DNA minikit (Qiagen) as described previously (40), with the following modifications: prior to the addition of the enzymatic lysis buffer, samples underwent a freeze-thaw step at –80°C for 15 min, and DNA was eluted in 100 μl of nuclease-free H_2_O (Ambion), with two elution steps of 50 μl each.

### Real-time PCR on isolates.

Real-time PCR assays were performed on isolate DNA normalized to a concentration of 1 ng/μl. Real-time PCR assays targeting the *lytA*, *piaB*, and *bguR* genes were performed using the same conditions and primer and probe concentrations as the qPCR assays. Isolates were considered positive when the *C_T_* was <30.

### MALDI-TOF MS.

All OP isolates were identified by MALDI-TOF MS using the Vitek MS (bioMérieux) system according to the manufacturer’s instructions. Following an overnight culture of the isolates on HBA, approximately four colonies (i.e., a visible amount of bacterial growth) were applied to the Vitek MS-DS target slide (bioMérieux) using a sterile toothpick. Immediately after application to the slide, 1 μl of Vitek MS-CHCA matrix solution (bioMérieux) was added to the bacteria on the slide. After allowing the matrix solution to air dry, the slide was loaded into the Vitek MS machine. Following run completion, results were analyzed using MYLA software (version 4; bioMérieux).

### S. pneumoniae isolate identification.

An isolate was identified as S. pneumoniae if at least three of the following results were obtained: optochin sensitivity, bile solubility, a MALDI-TOF MS result of S. pneumoniae, and *lytA* real-time PCR positivity. Where only two of the four tests were indicative of S. pneumoniae, the StrepID component of microarray, based on detection of species-specific genes, was used to discriminate between S. pneumoniae and non-S. pneumoniae. Where none or one of the tests was indicative of S. pneumoniae, an isolate was considered non-S. pneumoniae.

### Serotyping by latex agglutination and Quellung reaction.

Latex agglutination was performed using reagents produced in-house (41) with Statens Serum Institute (SSI) antisera on a sweep of growth from the sample culture plate (latex sweep) or an isolate (latex agglutination) (39, 55). Quellung testing was performed as previously described (42) using SSI antisera on isolates for which pneumococcal serotypes were detected using latex agglutination. Reactions were visualized using a microscope at a magnification of ×400.

### Serotyping by mPCR.

Serotyping multiplex PCR (mPCR) was performed using the method described by Carvalho et al. (43) and on the Centers for Disease Control and Prevention (CDC) *Streptococcus* Laboratory PCR Deduction of Pneumococcal Serotypes website (https://www.cdc.gov/streplab/pcr.html; accessed 14 July 2015) using 5 ng/μl of template DNA. PCR products were examined for size using an Agilent 2100 Bioanalyzer instrument (Agilent Technologies) and also run on an E-Gel *48* 2% agarose gel (Invitrogen) according to the manufacturer’s instructions. Results from both the E-Gel and Bioanalyzer methods were compared, and only products detected by both methods were considered true products. Products within 30 bp of an expected serotype product size were considered positive for the serotype.

### Whole-genome sequencing.

Two isolates which were not pneumococcus but yielded a pneumococcal serotype (using both latex agglutination and microarray) underwent whole-genome sequencing to identify any pneumococcal-like *cps* regions. Isolates FVEP-002-002-03 and FVEP-002-080-02 were sequenced using Illumina MiSeq V2 reagent kits (2 × 150 bp) on the MiSeq platform (Illumina). Sequence reads were down-sampled so that the assemblies would have approximately 150× coverage. Sequence assembly was performed using SPAdes version 3.5.0 (44). Assembled contigs were then submitted to the RAST server for annotation (45). Species identification was done using a combination of MetaPhlAN v2.0 (46), Kraken (47), and eMLSA (48), plus using 16S sequences from the isolates with 16S SILVA Incremental Aligner (SINA) version 1.2.11 (49), Greengenes BLAST (50), and NCBI BLAST (51). Capsule genes were identified following RAST annotation and were aligned against 33F and 19B reference sequences (GenBank accession numbers AJ006986.1 and CR931676.1, respectively); sequences that aligned poorly were checked by NCBI BLAST. Figures for aligned sequences were generated using easyfig version 2.2.2 (52).

### Data analysis.

Data were analyzed and graphs were generated using GraphPad Prism version 7.02 and R version 3.2.4. A *P* value of <0.05 was considered statistically significant. For differences in categorical data, Fisher’s exact test was used. When evaluating qPCR assays (alone or in combination), samples found to contain pneumococci following culture amplification and DNA microarray were considered true positives. The sensitivity, specificity, and positive predictive value (PPV) of assays were determined.

### Data availability.

The bacterial genomes for FVEP-002-002-03 and FVEP-002-080-02 analyzed during the current study are available in the NCBI Sequence Read Archive under accession number PRJNA590329 (https://www.ncbi.nlm.nih.gov/bioproject/590329). Other data and materials used are available upon reasonable request but may require ethical approval from the Fiji National Health Research and Ethics Review Committee and the University of Melbourne Health Sciences Human Ethics Subcommittee.

10.1128/mSphere.00478-20.7 TABLE S6 Comparison of results for 250 OP samples for pneumococcal targets alone and in combination using *C_T_* of < 35 as a cutoff. Download Table S6, DOCX file, 0.01 MB. Copyright © 2020 Boelsen et al. 2020 Boelsen et al. This content is distributed under the terms of the Creative Commons Attribution 4.0 International license.

10.1128/mSphere.00478-20.8 TABLE S7 Comparison of results for 250 OP samples for pneumococcal targets using a sequential screening approach. Download Table S7, DOCX file, 0.01 MB. Copyright © 2020 Boelsen et al. 2020 Boelsen et al. This content is distributed under the terms of the Creative Commons Attribution 4.0 International license.

10.1128/mSphere.00478-20.9 TABLE S8 Summary of the characterization of 91 isolates (derived from a subset of OP samples) using various identification and serotyping tests. Download Table S8, DOCX file, 0.01 MB. Copyright © 2020 Boelsen et al. 2020 Boelsen et al. This content is distributed under the terms of the Creative Commons Attribution 4.0 International license.

10.1128/mSphere.00478-20.10 TABLE S9 Full characterization of 91 isolates (derived from a subset of OP samples) using various identification and serotyping tests. Download Table S9, DOCX file, 0.04 MB. Copyright © 2020 Boelsen et al. 2020 Boelsen et al. This content is distributed under the terms of the Creative Commons Attribution 4.0 International license.
