# Supplementary figures and images for: The Challenges of Using Oropharyngeal Samples To Measure Pneumococcal Carriage in Adults
Source: mSphere. 2020 Jul 29;5(4):e00478-20. doi: 10.1128/mSphere.00478-20 (PMC7392543; doi:10.1128/mSphere.00478-20)

**b**

**a**


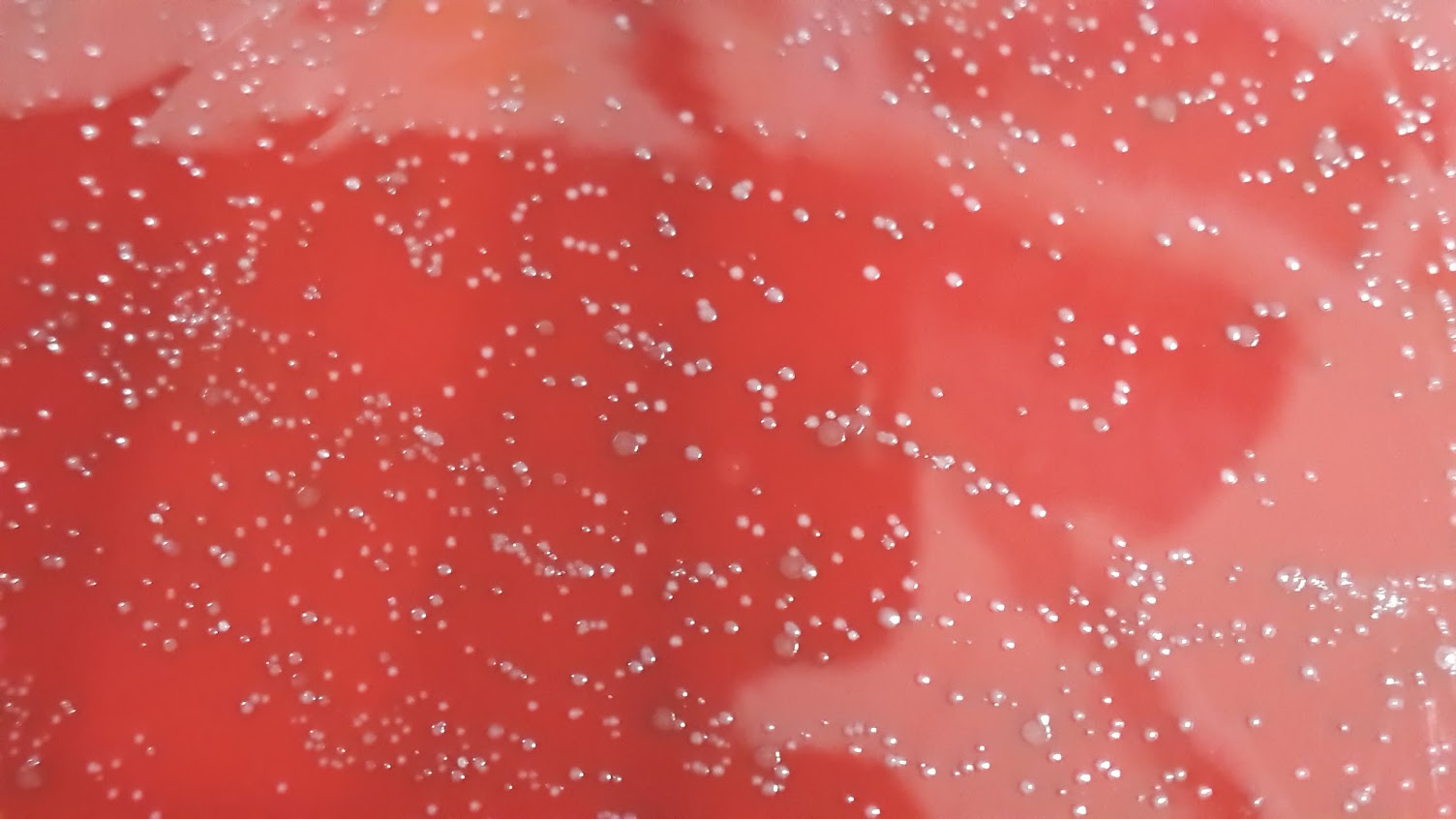

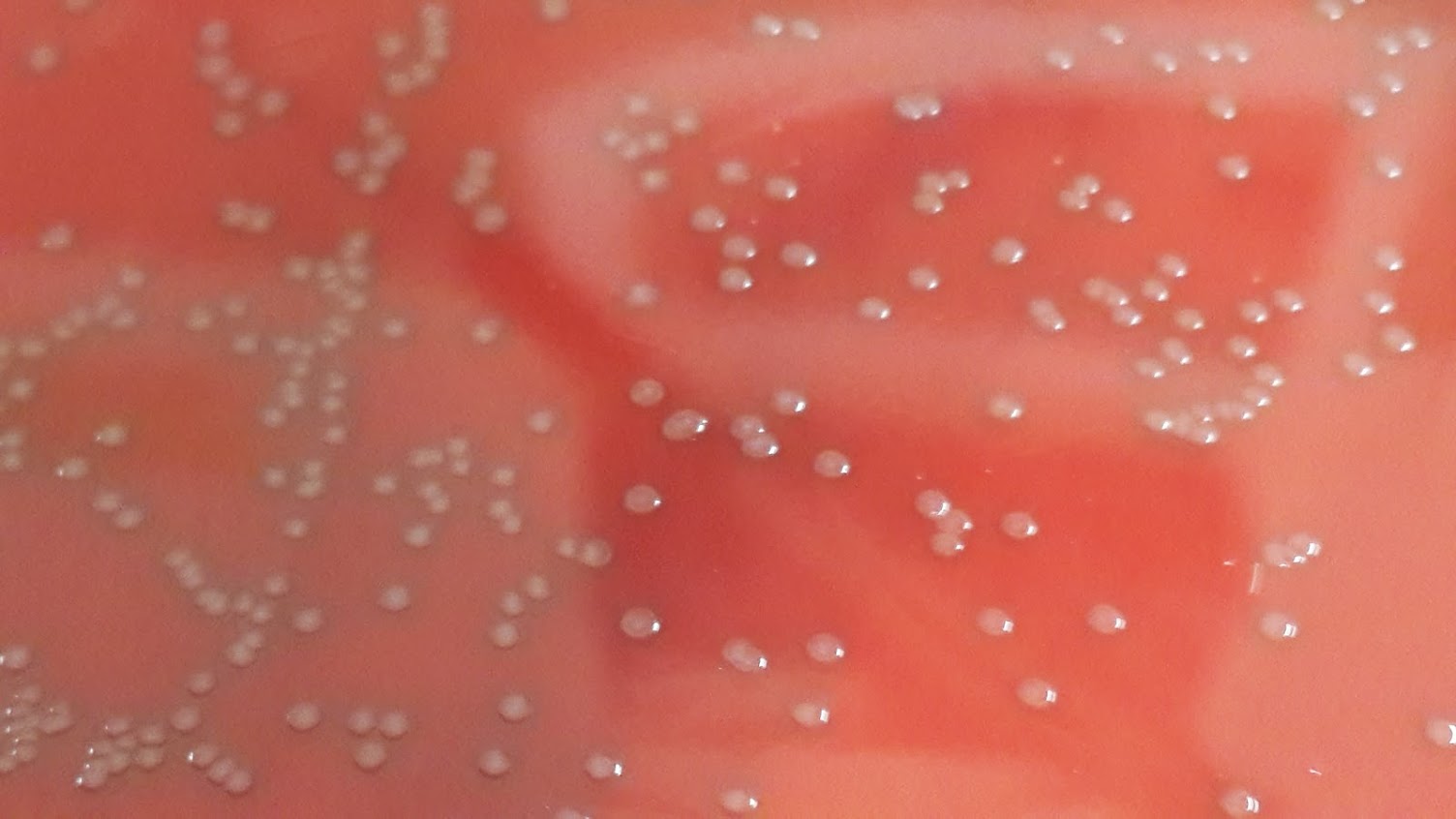


**FIGURE S1**

Supplement: FIG S1 [file mSphere.00478-20-sf001.docx]
