# Supplementary material for: The Challenges of Using Oropharyngeal Samples To Measure Pneumococcal Carriage in Adults
Source: mSphere. 2020 Jul 29;5(4):e00478-20. doi: 10.1128/mSphere.00478-20 (PMC7392543; doi:10.1128/mSphere.00478-20)
Supplement: TABLE S1 [file mSphere.00478-20-st001.docx]

**TABLE S1**

| Participants | 250 |
| --- | --- |
| Median age (IQR) | 31 (25-38) |
| Sex |  |
| Women | 232 (93%) |
| Men | 18 (7%) |
| Ethnicity |  |
| iTaukei | 156 (62%) |
| FID | 93 (37%) |
| Other | 1 (<1%) |
| Residential Location |  |
| Urban | 80 (32%) |
| Peri-urban | 52 (21%) |
| Rural | 118 (47%) |
| Median number of children under 5 years in the household (IQR) | 1 (1-2) |
| Exposure to household cigarette smoke | 141 (56%) |
| Symptoms of URTI | 55 (22%) |
| Antibiotic use in the past fortnight | 19 (8%) |
| Poverty^a^ | 151 (66%) |

Data are n (%), unless other specified; IQR, interquartile range; FID, Fijians of Indian Descent; URTI, upper respiratory tract infection; ^a^household income was not reported by 29 participants, poverty defined as weekly family income below the basic needs poverty line (<FJ$175 per week)
