# Supplementary material for: The Challenges of Using Oropharyngeal Samples To Measure Pneumococcal Carriage in Adults
Source: mSphere. 2020 Jul 29;5(4):e00478-20. doi: 10.1128/mSphere.00478-20 (PMC7392543; doi:10.1128/mSphere.00478-20)
Supplement: TABLE S2 [file mSphere.00478-20-st002.docx]

**TABLE S2**

| **Gene ID^a^** | **Gene** | **Product** | **Reference** |
| --- | --- | --- | --- |
| SP_1032  (SP_RS05120) | *piaA* | iron-compound ABC transporter, iron compound binding protein | (1) |
| SP_1033  (SP_RS05125) | *piaB* | iron-compound ABC transporter, permease protein | (2)^b^ |
| SP_2038  (SP_RS10305) | *ulaA* | PTS ascorbate transporter subunit IIC | (3) |
| SP_0049  (SP_RS00290) | *vanZ* | teicoplanin resistance protein | Microarray |
| SP_0137  (SP_RS00700) | - | ABC transporter ATP-binding protein | Microarray |
| SP_2020  (SP_RS10220) | *bguR* | GntR family transcriptional regulator | Microarray |
| SP_2167  (SP_RS11055) | *fucK* | L-fuculose kinase | Microarray |

^a^Gene ID refers to TIGR4 genome (NCBI Reference Sequence: NC_003028), updated gene ID shown in brackets; ^b^initially referred to as *piaA*, later amended to *piaB* (4).

**References**

1. Whalan RH, Funnell SGP, Bowler LD, Hudson MJ, Robinson A, Dowson CG. 2006. Distribution and genetic diversity of the ABC transporter lipoproteins PiuA and PiaA within *Streptococcus pneumoniae* and related streptococci. J Bacteriol 188:1031–1038.

2. Trzciński K, Bogaert D, Wyllie A, Chu MLJN, van der Ende A, Bruin JP, van den Dobbelsteen G, Veenhoven RH, Sanders EAM. 2013. Superiority of trans-oral over trans-nasal sampling in detecting *Streptococcus pneumoniae* colonization in adults. PLoS One 8:e60520.

3. Prère MF, Fayet OA. 2011. A specific polymerase chain reaction test for the identification of *Streptococcus pneumoniae*. Diagn Microbiol Infect Dis 70:45–53.

4. Wyllie AL, Wijmenga-Monsuur AJ, Van Houten MA, Bosch AATM, Groot JA, Van Engelsdorp Gastelaars J, Bruin JP, Bogaert D, Rots NY, Sanders EAM, Trzciński K. 2016. Molecular surveillance of nasopharyngeal carriage of *Streptococcus pneumoniae* in children vaccinated with conjugated polysaccharide pneumococcal vaccines. Sci Rep 6:23809.
