# Supplementary material for: The Challenges of Using Oropharyngeal Samples To Measure Pneumococcal Carriage in Adults
Source: mSphere. 2020 Jul 29;5(4):e00478-20. doi: 10.1128/mSphere.00478-20 (PMC7392543; doi:10.1128/mSphere.00478-20)
Supplement: TABLE S3 [file mSphere.00478-20-st003.docx]

**TABLE S3**

| **Gene**  **(TIGR4 ID)** | **Product size** | **Primer** | **Sequence (5’->3’)** |
| --- | --- | --- | --- |
| vanZ  (SP_0049) | 167 bp | Forward | GAATAGTCGGGACAGGTTTCT |
|  |  | Reverse | TCTAGTCTGTGATTTGAACACTCT |
| (SP_0137) | 153 bp | Forward | AGGCGCTATTAGCTTCTTTCTC |
|  |  | Reverse | CCTAATTCACCTAGCGCTGTAA |
| *bguR*  (SP_2020) | 115 bp | Forward | AGTTTGCCTGTAGTCGAATGA |
|  |  | Reverse | TTTGAGCTGCCACGAGAG |
| *fucK*  (SP_2167) | 201 bp | Forward | CGCGATTGTCTCAGTACCTAA |
|  |  | Reverse | CGTCTTAGTTCCTCTATGATCCAC |
