# Supplementary material for: The Challenges of Using Oropharyngeal Samples To Measure Pneumococcal Carriage in Adults
Source: mSphere. 2020 Jul 29;5(4):e00478-20. doi: 10.1128/mSphere.00478-20 (PMC7392543; doi:10.1128/mSphere.00478-20)
Supplement: TABLE S5 [file mSphere.00478-20-st005.docx]

**TABLE S5**

| ID | Species, serotype^ | *lytA* real-time PCR | *piaB* real-time PCR | *bguR* real-time PCR |
| --- | --- | --- | --- | --- |
| PMP6 | *S. pneumoniae*, 5 | + | + | + |
| PMP7 | *S. pneumoniae*, 19F | + | + | + |
| PMP85 | *S. pneumoniae*, 23F | + | + | + |
| PMP849 | *S. pneumoniae*, 1 | + | + | + |
| PMP1064 | *S. pneumoniae*, 22A | + | + | + |
| 0173-01 | *S. pneumoniae*, NT | + | + | + |
| 001-009-01 | *S. pseudopneumoniae* | - | - | - |
| PMP1300 | *S. pseudopneumoniae* | - | - | - |
| PMP16 | *S. mitis* | - | - | - |
| PMP933 | *S. mitis* | - | - | - |
| PMP934 | *S. mitis* | - | - | - |
| PMP1010 | *S. salivarius* | - | - | - |
| PMP935 | *S. mutans* | - | - | - |
| PMP1056 | *S. oralis* | - | - | - |
| PMP1301 | *S. infantis* | - | - | - |
| PMP1000 | *S. pyogenes* | - | - | - |
| PMP1057 | *S. gordonii* | - | - | - |
| PMP1035 | *S. bovis* | - | - | - |
| PMP1051 | *S. dysgalactiae* | - | - | - |
| PMP994 | *S. agalactiae* | - | - | - |
| PMP1053 | *S. sobrinus* | - | - | - |
| PMP936 | *S. sanguinis* | - | - | - |
| PMP1049 | *S. anginosus* | - | - | - |
| PMP1059 | *S. vestibularis* | - | - | - |
| PMP1302 | *S. peroris* | - | - | - |
| PMP1303 | *S. australis* | - | - | - |
| PMP1304 | *S. australis* | - | - | - |
| PMP1305 | *S. oligofermantans* | - | - | - |
| PMP1306 | *S. cristatus* | - | - | - |
| PMP1307 | *S. sinensis* | - | - | - |

+, positive Ct<30; –, negative Ct≥30.
