# Supplementary material for: The Challenges of Using Oropharyngeal Samples To Measure Pneumococcal Carriage in Adults
Source: mSphere. 2020 Jul 29;5(4):e00478-20. doi: 10.1128/mSphere.00478-20 (PMC7392543; doi:10.1128/mSphere.00478-20)
Supplement: TABLE S6 [file mSphere.00478-20-st006.docx]

**TABLE S6**

| **Target gene/s^a^** | **True positives (n)** | **False positives (n)** | **False negatives (n)** | **True Negatives (n)** | **PPV^a^ (%)** | | **Specificity (%)** | **Sensitivity (%)** |
| --- | --- | --- | --- | --- | --- | --- | --- | --- |
| *lytA* | 7 | 2 | 4 | 237 | | 78 | 99 | 64 |
| *bguR* | 4 | 5 | 7 | 234 | | 44 | 98 | 36 |
| *piaB* | 2 | 2 | 9 | 237 | | 50 | 99 | 18 |
| *lytA* + *bguR* | 4 | 1 | 7 | 238 | | 80 | 100 | 36 |
| *lytA* + *piaB* | 2 | 1 | 9 | 238 | | 67 | 100 | 18 |
| *bguR* + *piaB* | 2 | 1 | 9 | 238 | | 67 | 100 | 18 |
| *lytA* + *bguR* + *piaB* | 2 | 1 | 9 | 238 | | 67 | 100 | 18 |

A positive is defined as any Ct <35; ^a^Positive predictive value (PPV), specificity, and sensitivity were compared to DNA microarray, the study gold standard for pneumococcal-positive samples.
