# Supplementary material for: The Challenges of Using Oropharyngeal Samples To Measure Pneumococcal Carriage in Adults
Source: mSphere. 2020 Jul 29;5(4):e00478-20. doi: 10.1128/mSphere.00478-20 (PMC7392543; doi:10.1128/mSphere.00478-20)
Supplement: TABLE S7 [file mSphere.00478-20-st007.docx]

**TABLE S7**

| **Target gene/s** | **True positives**  **(n)** | **False positives**  **(n)** | **False negatives**  **(n)** | **True Negatives**  **(n)** | **PPV^b^ (%)** | **Specificity (%)** | **Sensitivity**  **(%)** |
| --- | --- | --- | --- | --- | --- | --- | --- |
| *lytA + bguR* or *piaB*^a^ | 10 | 17 | 1 | 222 | 37 | 93 | 91 |
| *bguR + lytA* or *piaB*^a^ | 10 | 16 | 1 | 223 | 38 | 93 | 91 |
| *piaB + lytA* or *bguR*^a^ | 9 | 13 | 2 | 226 | 41 | 95 | 82 |

PPV, positive predictive value; ^a^Only samples with Ct<40 by the first target were further tested with the other two targets, a positive sample was defined as Ct<40 for at least two targets; ^b^Positive predictive value (PPV), specificity, and sensitivity were compared to DNA microarray, the study gold standard for pneumococcal-positive samples.
