# Supplementary material for: The Challenges of Using Oropharyngeal Samples To Measure Pneumococcal Carriage in Adults
Source: mSphere. 2020 Jul 29;5(4):e00478-20. doi: 10.1128/mSphere.00478-20 (PMC7392543; doi:10.1128/mSphere.00478-20)
Supplement: TABLE S8 [file mSphere.00478-20-st008.docx]

**TABLE S8**

| **Test** | **All isolates**  n=91 | ***S. pneumoniae* isolates**  n=3 | **non-*S. pneumoniae* isolates only**  n=88 |
| --- | --- | --- | --- |
| ***lytA* real-time PCR** |  |  |  |
| Positive | 3 (3%) | 3 (100%) | 0 (0 %) |
| Equivocal | 10 (11%) | 0 (0%) | 10 (11%) |
| Negative | 78 (86%) | 0 (0%) | 78 (89%) |
| **Optochin** |  |  |  |
| Sensitive | 3 (3%) | 3 (100%) | 0 (0%) |
| Intermediate | 1 (1%) | 0 (0%) | 1 (1%) |
| Resistant | 87 (96%) | 0 (0%) | 87 (99%) |
| **MALDI-TOF MS** |  |  |  |
| ID obtained | 80 (88%) | 3 (100%) | 77 (88%) |
| No ID obtained | 11 (12%) | 0 (0%) | 11 (13%) |
| **Bile solubility** |  |  |  |
| Soluble | 5 (5%) | 3 (100%) | 2 (2%) |
| Insoluble | 58 (64%) | 0 (0%) | 58 (66%) |
| Not readable^ | 28 (31%) | 0 (0%) | 28 (32%) |
| **Latex Agglutination** |  |  |  |
| Serotyping result | 13 (14%) | 3 (100%) | 10 (11%) |
| Negative* | 45 (49%) | 0 (0%) | 45 (51%) |
| Not readable^ | 33 (36%) | 0 (0%) | 33 (38%) |
| **Microarray** |  |  |  |
| Serotyping result | 39 (43%) | 1 (33%) | 38 (43%) |
| Negative* | 3 (3%) | 0 (0%) | 3 (3%) |
| Not tested | 49 (54%) | 2 (67%) | 47 (53%) |

‘Serotyping result’ indicates a serotyping result was obtained, this includes pseudoserotypes from non-pneumococcal species; *Indicates no serotyping result was obtained; ^Bacterial culture did not emulsify in saline or only partially emulsified in saline rendering the sample ‘not-readable’.
