# Supplementary material for: The Challenges of Using Oropharyngeal Samples To Measure Pneumococcal Carriage in Adults
Source: mSphere. 2020 Jul 29;5(4):e00478-20. doi: 10.1128/mSphere.00478-20 (PMC7392543; doi:10.1128/mSphere.00478-20)
Supplement: TABLE S9 [file mSphere.00478-20-st009.docx]

**TABLE S9**

| **Sample** | **Isolate** | **Optochin** | **Bile test** | ***lytA* qPCR** | | **MALDI-TOF MS** | **Latex agglutination** | **Microarray** | | | **Multiplex PCR** |
| --- | --- | --- | --- | --- | --- | --- | --- | --- | --- | --- | --- |
|  |  |  |  | **Ct value** | **Result** |  |  | **serotyping** | **StrepID** | **PathID** |  |
| FVEP-002-002 | 01 | Resistant | insoluble | No Ct | negative | *S. salivarius* ssp. salivarius | 19B; 12 | 36/7F-like* | saliva | SP-1/5 | not tested |
| FVEP-002-002 | 02 | Resistant | insoluble | No Ct | negative | *S. dysgalactiae* | negative | 7F-like* | negative | SP-1/5 | not tested |
| FVEP-002-002 | 03 | Resistant | insoluble | No Ct | negative | no identification | 33F; 35 | 33F-like* | oralis | SP-3/5 | not tested |
| FVEP-002-002 | 04 | Resistant | not readable | No Ct | negative | *S. pneumoniae* | 19B | 19B-like* | mitis; infant | SP-2/5 | not tested |
| FVEP-002-002 | 05 | Resistant | insoluble | No Ct | negative | *S. mitis*/*S. oralis* | negative | not tested | not tested | not tested | not tested |
| FVEP-002-002 | 06 | Resistant | not readable | No Ct | negative | *S. mitis*/*S. oralis* | not tested | not tested | not tested | not tested | not tested |
| FVEP-002-002 | 07 | Resistant | insoluble | No Ct | negative | *Pseudomonas fluorescens* | negative | 14-like* | oralis | SP-3/5 | not tested |
| FVEP-002-002 | 08 | Resistant | not readable | No Ct | negative | *S. mitis*/*S. oralis* | not tested | not tested | not tested | not tested | not tested |
| FVEP-002-002 | 09 | Resistant | not readable | No Ct | negative | *S. mitis*/*S. oralis* | not tested | not tested | not tested | not tested | not tested |
| FVEP-002-488 | 01 | Resistant | insoluble | No Ct | negative | no identification | negative | 36/33A/39-like* | infant | SP-2/5 | not tested |
| FVEP-002-488 | 02 | Resistant | not readable | No Ct | negative | *S. mitis*/*S. oralis* | not tested | 48-like* | infant | SP-2/5 | not tested |
| FVEP-002-488 | 03 | Resistant | not readable | No Ct | negative | *S. mitis*/*S. oralis* | not tested | NT4a*(54%)+43-like*(46%) | mitis; oralis | SP-3/5 | not tested |
| FVEP-002-488 | 04 | Resistant | insoluble | No Ct | negative | *S. parasanguinis* | negative | 10C/21-like* | negative | SP-1/5 | not tested |
| FVEP-002-488 | 05 | Resistant | insoluble | No Ct | negative | *Rothia mucilaginosa* | negative | negative | negative | SP-0/5 | not tested |
| FVEP-002-488 | 06 | Resistant | insoluble | No Ct | negative | *S. salivarius* ssp. salivarius | 19B; 12 | 7F/36-like* | saliva | SP-1/5 | not tested |
| FVEP-002-004 | 01 | Resistant | insoluble | No Ct | negative | *S. mitis*/*S. oralis* | negative | not tested | not tested | not tested | not tested |
| FVEP-002-004 | 02 | Resistant | not readable | No Ct | negative | no identification | negative | 10C/21/7F-like* | saliva | SP-1/5 | not tested |
| FVEP-002-004 | 03 | Resistant | insoluble | No Ct | negative | *S. mitis*/*S. oralis* | negative | not tested | not tested | not tested | not tested |
| FVEP-002-004 | 04 | Resistant | insoluble | No Ct | negative | *S. mitis*/*S. oralis* | negative | not tested | not tested | not tested | not tested |
| FVEP-002-004 | 05 | Resistant | insoluble | No Ct | negative | *S. mitis*/*S. oralis* | negative | not tested | not tested | not tested | not tested |
| FVEP-002-084 | 01 | Resistant | insoluble | No Ct | negative | *S. mitis*/*S. oralis* | negative | not tested | not tested | not tested | not tested |
| FVEP-002-084 | 02 | Resistant | insoluble | No Ct | negative | *S. mitis*/*S. oralis* | negative | not tested | not tested | not tested | not tested |
| FVEP-002-084 | 03 | Resistant | insoluble | No Ct | negative | *Paracoccus yeei* | negative | NT4a*(55%)+36-like*(45%) | mitis | SP-3/5 | not tested |
| FVEP-002-084 | 04 | Resistant | insoluble | No Ct | negative | *S. mitis*/*S. oralis* | negative | not tested | not tested | not tested | not tested |
| FVEP-002-084 | 05 | Intermediate | not readable | No Ct | negative | *S. mitis*/*S. oralis* | not tested | not tested | not tested | not tested | not tested |
| FVEP-002-084 | 06 | Resistant | insoluble | No Ct | negative | *S. mitis*/*S. oralis* | negative | not tested | not tested | not tested | not tested |
| FVEP-002-084 | 07 | Resistant | insoluble | No Ct | negative | *S. salivarius* ssp. salivarius | 19B; 12 | 36-like* | saliva | SP-1/5 | not tested |
| FVEP-002-084 | 08 | Resistant | insoluble | No Ct | negative | *S. mitis*/*S. oralis* | negative | not tested | not tested | not tested | not tested |
| FVEP-002-078 | 01 | Resistant | insoluble | No Ct | negative | *S. mitis*/*S. oralis* | 35 - | 7F/33A-like* | oralis | SP-3/5 | not tested |
| FVEP-002-078 | 02 | Resistant | insoluble | No Ct | negative | *S. mitis*/*S. oralis* | negative | not tested | not tested | not tested | not tested |
| FVEP-002-078 | 03 | Resistant | insoluble | No Ct | negative | no identification | negative | not tested | not tested | not tested | not tested |
| FVEP-002-078 | 04 | Resistant | not readable | No Ct | negative | S. salivarius ssp. salivarius | not tested | not tested | not tested | not tested | not tested |
| FVEP-002-078 | 05 | Resistant | insoluble | No Ct | negative | *S. parasanguinis* | negative | not tested | not tested | not tested | not tested |
| FVEP-002-078 | 06 | Resistant | insoluble | No Ct | negative | *S. mitis*/*S. oralis* | negative | not tested | not tested | not tested | not tested |
| FVEP-002-078 | 07 | Resistant | not readable | No Ct | negative | no identification | not tested | not tested | not tested | not tested | not tested |
| FVEP-002-078 | 08 | Resistant | insoluble | No Ct | negative | *S. anginosus* | negative | not tested | not tested | not tested | not tested |
| FVEP-002-078 | 09 | Resistant | insoluble | No Ct | negative | *S. mitis*/*S. oralis* | 35 - | 7F/21/33A-like*(65%)+NT4b*(35%) | oralis | SP-3/5 | not tested |
| FVEP-002-496 | 01 | Resistant | insoluble | No Ct | negative | *S. mitis*/*S. oralis* | negative | not tested | not tested | not tested | not tested |
| FVEP-002-496 | 02 | Resistant | not readable | No Ct | negative | *S. salivarius* ssp. salivarius | not tested | not tested | not tested | not tested | not tested |
| FVEP-002-496 | 03 | Resistant | insoluble | No Ct | negative | *S. mitis*/*S. oralis* | negative | not tested | not tested | not tested | not tested |
| FVEP-002-496 | 04 | Resistant | not readable | No Ct | negative | no identification | not tested | 36-like* | parasa | SP-1/5 | not tested |
| FVEP-002-496 | 05 | Resistant | insoluble | No Ct | negative | *S. parasanguinis* | negative | 10C/21-like* | parasa | SP-1/5 | not tested |
| FVEP-002-496 | 06 | Resistant | insoluble | No Ct | negative | no identification | negative | not tested | not tested | not tested | not tested |
| FVEP-002-496 | 07 | Resistant | insoluble | No Ct | negative | *S. mitis*/*S. oralis* | negative | not tested | not tested | not tested | not tested |
| FVEP-002-496 | 08 | Resistant | insoluble | No Ct | negative | *S. cristatus* | 19B | 24F/16F-like* | sangui | SP-1/5 | not tested |
| FVEP-002-496 | 09 | Resistant | insoluble | No Ct | negative | *S. salivarius* ssp. salivarius | 19B; 12 | 36-like* | saliva | SP-1/5 | not tested |
| FVEP-002-080 | 01 | Resistant | not readable | 38.28 | equivocal | *S. salivarius* ssp. salivarius | not tested | 7F-like* | saliva | SP-1/5 | not tested |
| FVEP-002-080 | 02 | Resistant | insoluble | No Ct | negative | *S. mitis*/*S. oralis* | 19B | 19B-like* | mitis; infant | SP-2/5 | not tested |
| FVEP-002-080 | 03 | Resistant | not readable | No Ct | negative | *S. mitis*/*S. oralis* | not tested | not tested | not tested | not tested | not tested |
| FVEP-002-080 | 04 | Resistant | insoluble | No Ct | negative | *S. mitis*/*S. oralis* | negative | not tested | not tested | not tested | not tested |
| FVEP-002-080 | 05 | Resistant | insoluble | No Ct | negative | *S. pneumoniae* | negative | NT4b* | mitis | SP-3/5 | not tested |
| FVEP-002-080 | 06 | Resistant | not readable | No Ct | negative | *S. mitis*/*S. oralis* | not tested | not tested | not tested | not tested | not tested |
| FVEP-002-080 | 07 | Resistant | insoluble | No Ct | negative | *S. parasanguinis* | negative | not tested | not tested | not tested | not tested |
| FVEP-002-080 | 08 | Resistant | not readable | No Ct | negative | *S. parasanguinis* | not tested | not tested | not tested | not tested | not tested |
| FVEP-002-080 | 09 | Resistant | not readable | No Ct | negative | *Rothia mucilaginosa* | not tested | negative | negative | SP-0/5 | not tested |
| FVEP-002-080 | 10 | Resistant | insoluble | No Ct | negative | *S. parasanguinis* | negative | not tested | not tested | not tested | not tested |
| FVEP-002-080 | 11 | Sensitive | soluble | 18.31 | positive | *S. pneumoniae* | 1 | not tested | not tested | not tested | not tested |
| FVEP-002-418 | 01 | Resistant | not readable | No Ct | negative | no identification | not tested | not tested | not tested | not tested | not tested |
| FVEP-002-418 | 02 | Resistant | not readable | 35.23 | equivocal | *S. salivarius* ssp. salivarius | not tested | 7F-like* | saliva | SP-1/5 | not tested |
| FVEP-002-418 | 03 | Resistant | insoluble | 37.55 | equivocal | *S. mitis*/*S. oralis* | negative | 16A-like* | oralis | SP-3/5 | not tested |
| FVEP-002-418 | 04 | Resistant | not readable | No Ct | negative | no identification | not tested | not tested | not tested | not tested | not tested |
| FVEP-002-418 | 05 | Resistant | insoluble | No Ct | negative | *S. mitis*/*S. oralis* | negative | not tested | not tested | not tested | not tested |
| FVEP-002-418 | 06 | Resistant | not readable | 37.22 | equivocal | *S. mitis*/*S. oralis* | not tested | 7F/36-like*(53%)+NT4a*(47%) | mitis | SP-3/5 | not tested |
| FVEP-002-418 | 07 | Resistant | insoluble | No Ct | negative | *S. mitis*/*S. oralis* | negative | not tested | not tested | not tested | not tested |
| FVEP-002-418 | 08 | Resistant | insoluble | No Ct | negative | *S. mitis*/*S. oralis* | negative | not tested | not tested | not tested | not tested |
| FVEP-002-418 | 09 | Resistant | insoluble | No Ct | negative | *S. anginosus* | negative | not tested | not tested | not tested | not tested |
| FVEP-002-460 | 01 | Resistant | insoluble | No Ct | negative | *S. mitis*/*S. oralis* | negative | 33A/21/10A-like* | oralis | SP-3/5 | 10A + 33F/33A/37 + 5 + 23B |
| FVEP-002-460 | 02 | Resistant | not readable | 37.97 | equivocal | *S. salivarius* ssp. salivarius | not tested | 7F-like* | saliva | SP-1/5 | 10A +13 |
| FVEP-002-460 | 03 | Sensitive | soluble | 17.61 | positive | *S. pneumoniae* | 35B | 35B | pneumo | SP-5/5 | 35B |
| FVEP-002-460 | 04 | Resistant | insoluble | No Ct | negative | *S. mitis*/*S. oralis* | negative | 20-like* | infant | SP-2/5 | 15A/15A + 31 + 20 |
| FVEP-002-460 | 05 | Resistant | not readable | 39.61 | equivocal | *S. mitis*/*S. oralis* | not tested | 36-like* | infant | SP-1/5 | 20 |
| FVEP-002-460 | 06 | Resistant | insoluble | No Ct | negative | *S. mitis*/*S. oralis* | negative | NT4b*(55%)+43/29-like*(45%) | mitis | SP-3/5 | 13 |
| FVEP-002-460 | 07 | Resistant | insoluble | 36.9 | equivocal | *S. mitis*/*S. oralis* | negative | NT4b* | mitis | SP-3/5 | 6A/6B/6C/6D + 15A/15F +10A |
| FVEP-002-460 | 08 | Resistant | insoluble | No Ct | negative | *S. anginosus* | negative | 7F/21-like* | angino | SP-1/5 | 6A/6B/6C/6D + 22F/22A + 35F/47F + 6C/6D |
| FVEP-002-460 | 09 | Resistant | insoluble | No Ct | negative | no identification | negative | 10C/21-like* | oralis | SP-2/5 | 7F/7A + 9V/9A + 1 + 35A/35C/42 + 2 |
| FVEP-002-460 | 10 | Resistant | insoluble | No Ct | negative | *S. parasanguinis* | negative | 7F/36-like* | parasa | SP-1/5 | 22F/22A + 7F/7A + 5 |
| FVEP-002-422 | 01 | Resistant | insoluble | No Ct | negative | *S. mitis*/*S. oralis* | not tested | not tested | not tested | not tested | not tested |
| FVEP-002-422 | 02 | Sensitive | soluble | 18.26 | positive | *S. pneumoniae* | 15B | not tested | not tested | not tested | not tested |
| FVEP-002-422 | 03 | Resistant | not readable | 39.57 | equivocal | *S. salivarius* ssp. salivarius | not tested | 7F-like* | saliva | SP-1/5 | not tested |
| FVEP-002-422 | 04 | Resistant | insoluble | No Ct | negative | *S. parasanguinis* | not tested | not tested | not tested | not tested | not tested |
| FVEP-002-422 | 05 | Resistant | not readable | No Ct | negative | *S. pneumoniae* | not tested | NT4a*(65%)+7F-like*(35%) | mitis | SP-3/5 | not tested |
| FVEP-002-422 | 06 | Resistant | insoluble | No Ct | negative | *S. parasanguinis* | not tested | not tested | not tested | not tested | not tested |
| FVEP-002-422 | 07 | Resistant | not readable | No Ct | negative | *S. mitis*/*S. oralis* | not tested | not tested | not tested | not tested | not tested |
| FVEP-002-486 | 01 | Resistant | not readable | 38.43 | equivocal | *S. salivarius* ssp. salivarius | not tested | 7F-like*(67%)+NT4b*(20%)+21-like*(13%) | saliva; mitis | SP-3/5 | not tested |
| FVEP-002-486 | 02 | Resistant | not readable | 39.49 | equivocal | *Rothia mucilaginosa* | not tested | NT4b* | mitis | SP-3/5 | not tested |
| FVEP-002-486 | 03 | Resistant | insoluble | No Ct | negative | *S. parasanguinis* | not tested | not tested | not tested | not tested | not tested |
| FVEP-002-486 | 04 | Resistant | insoluble | No Ct | negative | *S. parasanguinis* | not tested | not tested | not tested | not tested | not tested |
| FVEP-002-486 | 05 | Resistant | soluble | No Ct | negative | *S. mitis/S. oralis* | negative | not tested | not tested | not tested | not tested |
| FVEP-002-486 | 06 | Resistant | insoluble | No Ct | negative | *S. mitis/S. oralis* | not tested | not tested | not tested | not tested | not tested |
| FVEP-002-486 | 07 | Resistant | insoluble | No Ct | negative | *S. parasanguinis* | not tested | not tested | not tested | not tested | not tested |
| FVEP-002-486 | 08 | Resistant | soluble | No Ct | negative | no identification | negative | negative | negative | SP-0/5 | not tested |

StrepID microarray: saliva, *Streptococcus salivarius*; oralis, *Streptococcus oralis*; mitis, *Streptococcus mitis*; infant, *Streptococcus infantis*; parasa, *Streptococcus parasanguinis*; sangui, *Streptococcus sanguinis*; pneumo, *Streptococcus pneumoniae*; angina, *Streptococcus anginosus*
